# Perilipin1 inhibits *Nosema bombycis* proliferation by promoting *Domeless-* and *Hop-*mediated JAK-STAT pathway activation in *Bombyx mori*

**DOI:** 10.1128/spectrum.03671-23

**Published:** 2024-05-01

**Authors:** Yaping Su, Qingsheng Qu, Junling Li, Zhenghao Han, Yujia Fang, Billong Laura Flavorta, Zhenwei Jia, Qiong Yu, Yiling Zhang, Ping Qian, Xudong Tang

**Affiliations:** 1 Jiangsu Key Laboratory of Sericultural and Animal Biotechnology, Jiangsu University of Science and Technology, Zhenjiang, China; 2 Key Laboratory of Silkworm and Mulberry Genetic Improvement, Ministry of Agriculture and Rural Affairs, Sericultural Research Institute, Chinese Academy of Agricultural Sciences, Zhenjiang, China; Weill Cornell Medicine, New York, New York, USA

**Keywords:** perilipin, *Nosema bombycis*, microsporidia, silkworm, JAK-STAT pathway, LSD-1

## Abstract

Lipid droplets (LDs) are dynamic organelles that participate in the regulation of lipid metabolism and cellular homeostasis inside of cells. LD-associated proteins, also known as perilipins (PLINs), are a family of proteins found on the surface of LDs that regulate lipid metabolism, immunity, and other functions. In silkworms, pébrine disease caused by infection by the microsporidian *Nosema bombycis* (*Nb*) is a severe threat to the sericultural industry. Although we found that *Nb* relies on lipids from silkworms to facilitate its proliferation, the relationship between PLINs and *Nb* proliferation remains unknown. Here, we found *Nb* infection caused the accumulation of LDs in the fat bodies of silkworm larvae. The characterized perilipin1 gene (*plin1*) promotes the accumulation of intracellular LDs and is involved in *Nb* proliferation. *plin1* is similar to *perilipin1* in humans and is conserved in all insects. The expression of *plin1* was mostly enriched in the fat body rather than in other tissues. Knockdown of *plin1* enhanced *Nb* proliferation, whereas overexpression of *plin1* inhibited its proliferation. Furthermore, we confirmed that *plin1* increased the expression of the *Domeless* and *Hop* in the JAK-STAT immune pathway and inhibited *Nb* proliferation. Taken together, our current findings demonstrate that *plin1* inhibits *Nb* proliferation by promoting the JAK-STAT pathway through increased expression of *Domeless* and *Hop*. This study provides new insights into the complicated connections among microsporidia pathogens, LD surface proteins, and insect immunity.

**IMPORTANCE:**

Lipid droplets (LDs) are lipid storage sites in cells and are present in almost all animals. Many studies have found that LDs may play a role in host resistance to pathogens and are closely related to innate immunity. The present study found that a surface protein of insect lipid droplets could not only regulate the morphological changes of lipid droplets but also inhibit the proliferation of a microsporidian pathogen *Nosema bombycis* (*Nb*) by activating the JAK-STAT signaling pathway. This is the first discovery of the relationship between microsporidian pathogen and insect lipid surface protein perilipin and insect immunity.

## INTRODUCTION

Lipid droplets (LDs) are dynamic organelles wrapped in a single layer of phospholipid molecules originating from the endoplasmic reticulum, which are responsible for the storage of neutral fats (such as triglycerides and cholesterol esters) and are the center of cellular lipid metabolism (1–3). Increasing evidence suggests that LDs not only serve as a source of energy in host physiological activities but are also involved in pathogen infection. The increasing of LDs in cytoplasm can be found in many pathogen-infected cells, including bacteria, parasites, and viruses, proving that infection-driven LD formation promotes innate immune activation and plays key roles in host–pathogen interactions (4).

LD-associated proteins, also known as perilipins (PLINs) are the most abundant proteins on the surface of LDs and regulate lipid storage and hydrolysis by binding to specific sites on the surface of LDs (5). PLINs were first discovered in the LDs of rat adipocytes by Greenberg in 1991 (6). To date, five different PLINs (PLIN1–5) have been identified in various human tissues (7–11). Both PLIN1 and PLIN2 are localized on the surface of LDs; however, they play opposite roles by recruiting different chaperone proteins from the cytoplasm to promote LD catabolism or synthesis, respectively. PLIN1 recruits hormone-sensitive lipase (HSL) to promote LD catabolism and reduce droplet size, whereas PLIN2 recruits adipose triglyceride lipase (ATGL) to promote LD synthesis and increase LD size (12). PLIN3, PLIN4, and PLIN5 are mainly localized in the cytoplasm and endoplasmic reticulum, and PLIN3 can compensate for PLIN2 in promoting LD stabilization when absent, as well as participate in intracellular transport to produce prostaglandin E2 (13). PLIN4 has been found to be associated with human adipocyte differentiation, whereas PLIN5 promotes LD stabilization and is involved in LD coupling to the mitochondria (14, 15).

Microsporidia is a class of obligate intracellular parasitic eukaryotes that exist widely in nature and can infect almost all animals, including invertebrates and vertebrates (16, 17). In the 1840s, Louis Pasteur studied the economically damaging silkworm disease pébrine, caused by infection by the microsporidium *Nosema bombycis* (*Nb*), which had caused great damage to European sericulture in the 19th century (18, 19). The discovery and naming of *Nb* are considered the beginning of microsporidia research (20). Microsporidia eject polar tubes to penetrate host cells at relatively short distances, and then the spore plasma is introduced into the host cytoplasm, where it metabolizes host nutrients for growth and reproduction (21). Microsporidian infection triggers a series of alterations in intracellular processes such as basic metabolism, ATP synthesis, protein and fat degradation, apoptosis, and autophagy (22–24). Some alterations may favor microsporidian proliferation, while others are pathways by which the host inhibits proliferation (25). Regarding intracellular lipid metabolism, the relationship between LDs and microsporidian proliferation remains unclear. We have previously found that *Nb* infection changes lipid metabolic homeostasis in *B. mori* and that *Nb* exploits the lipid metabolic system of silkworms to facilitate its own proliferation (26). In the present study, we investigated the characters of an LD surface protein plin1 and its effects on the proliferation of *Nb* in silkworms. We reveal a connection between microsporidia pathogens, perilipins, and insect immunity.

## RESULTS

### Clone and sequence analysis of *plin1*

**Fig 1:**
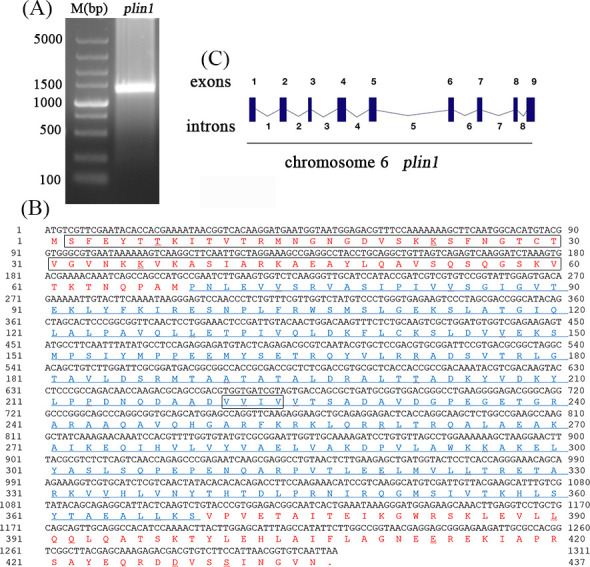
Cloning and sequence characteristics of *plin1*. (**A**) PCR of *plin1* with cDNA from the fat body tissues of the silkworm. The gel image shows the DL5000 DNA marker and the 1,311 bp PCR product of *plin1*. (**B**) CDS and protein sequences of *plin1*. The amino acid sequence from 69 to 369 codes for the perilipin superfamily (accession: cl03851, shown in blue). The missing amino acids (aa22–60 and aa222–225) in NCBI-annotated perilipin were framed. (**C**) Intron and exon distributions of *plin1* on chromosome 6 of the silkworm.

To explore the effects of *Nb* infection on silkworms, we previously examined the genes and proteins that were differentially expressed in the midgut and ovary of silkworm after *Nb* infection using transcriptomic and quantitative proteomic assays (23). In both data sets, plin1 (BGIBMGA013593 in SilkDB3.0) was differentially expressed. As such, we hypothesized that it may be related to *Nb* proliferation. Therefore, *plin1* (BGIBMGA013593) was cloned from cDNA derived from the silkworm fat body, and the sequence was analyzed. The results showed that the coding region of *plin1* was 1311 bp, encoding a protein containing 436 amino acids, which was consistent with PLIN1/LSD-1 from most *Lepidoptera* insects. A perilipin super family domain (69–369 aa, Accession:cl03851) of the plin1 protein could be found by NCBI Conserved Domains analysis (Fig. 1A and B). It has to be noted that NP_001040143.1 in NCBI and Q2F665_BOMMO in Uniprot are also annotated as the perilipin of silkworm. However, when compared with our cloned BGIBMGA013593, the NCBI- and Uniprot-annotated perilipins lack aa22–60 and aa222–225, respectively (Fig. 1B), and further experiments are needed to verify whether the shorter perilipin is indeed the isoform of *plin1*. According to the SilkDB3.0 genome database, the chromosomal location of *plin1* is on chromosome 6 of the silkworm. The coding region consists of nine exons and eight introns. (Fig. 1C). Comparison of *B.mori* PLIN1 with that of other species showed that PLIN1 had higher identity with that of *Pectinophora gossypiella*, *Hyposmocoma kahamanoa*, *Spodoptera frugiperda*, and *Manduca sexta* (with 86%, 79%, 77%, and 82% identity, respectively). Also, the plin1 and plin2 from different insects were collected to investigate the phylogenetic relationships of the insect *plins*, and the result showed that the insect *plins* can be divided quite clearly into two main branches (Fig. S1).

### Tissue and cellular distribution of *plin1*

**Fig 2:**
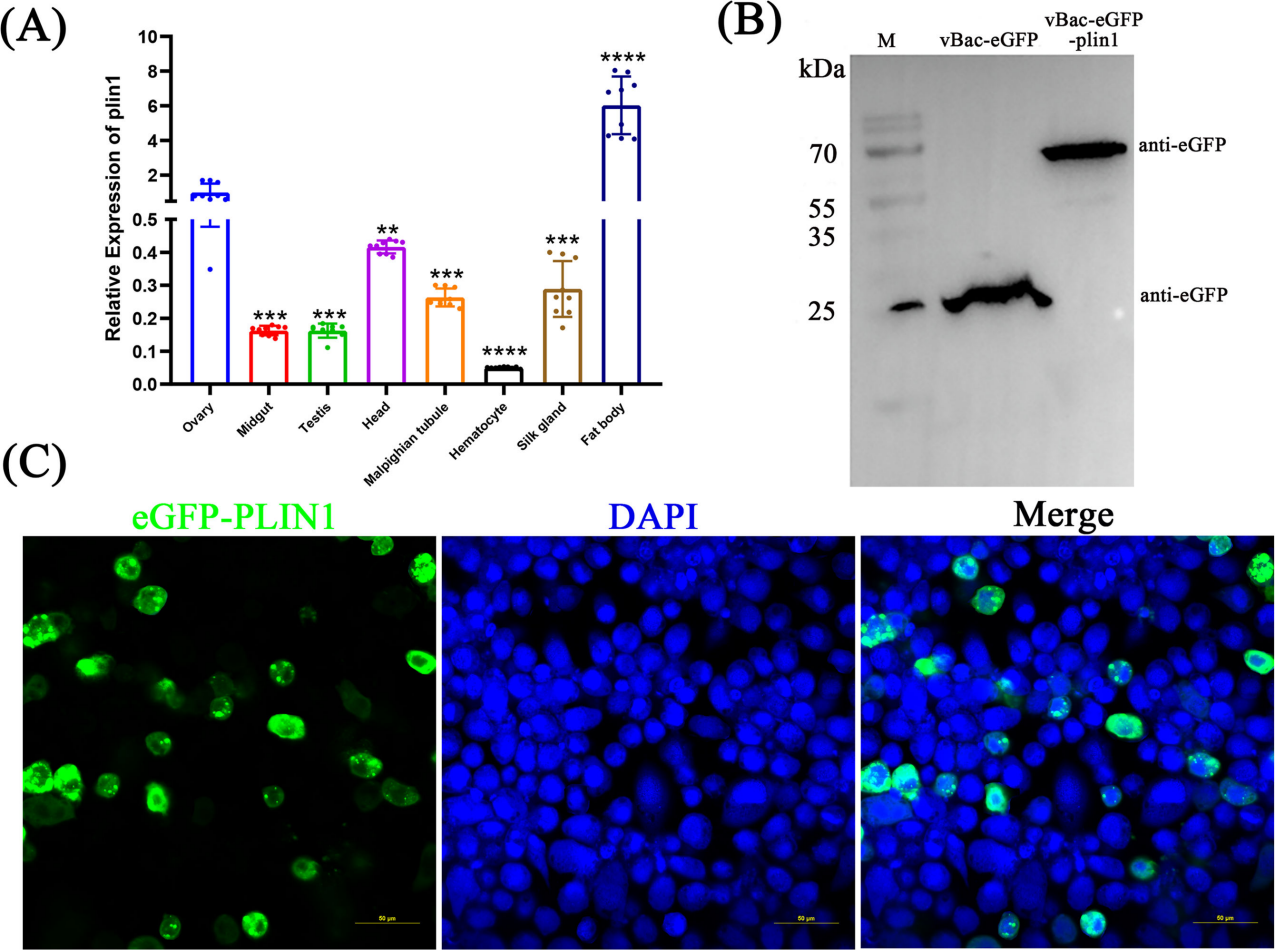
Expression of *plin1* in silkworm tissues and BmN cells. (**A**) The relative expression of plin1 was measured in the tissues of third day fifth instar larva of the silkworm and normalized to the ovary. The experiment was repeated three times. (**B**) Western blotting analysis of eGFP-plin1 with anti-GFP antibody. lane 1: marker: 180 kDa, lane 2: vBac-eGFP-infected cells. lane 3: vBac-eGFP-plin1-infected cells. (**C**) Subcellular localization of *plin1* in BmN cells. Error bars represent mean ± SD. One-way ANOVA or *t*-test, **P < 0.01; ****P < 0.0001. ns, not significant.

To investigate the expression of *plin1* in different tissues of silkworms, the expression of *plin1* in the ovaries, midgut, testis, head, Malpighian tubule, hematocytes, silk glands, and fat body of third day fifth instar silkworm larvae was detected using qPCR. The results showed that the expression of p*lin1* was highest in the fat body and lowest in hematocytes (Fig. 2A), which was consistent with the expression in the SilkDB3.0 database. *Plin1* is distributed in large quantities within the fat body, which is the primary site of LD storage and metabolism.

To confirm the cellular localization of *plin1* in BmN cells, *plin1* was fused and expressed using an eGFP-tag with the recombinant baculovirus vBac-eGFP-plin1. The results showed that the control virus-infected cells only expressed approximately 26 kDa of the eGFP protein, whereas in vBac-eGFP-plin1-infected cells, a band of 70 kDa was detected, consistent with the expected size of the eGFP–plin1 fusion protein, indicating that vBac-eGFP-plin1 was successfully constructed (Fig. 2B). The fluorescence signal of the eGFP–plin1 fusion protein was present mainly in the cytoplasm, which was also consistent with the distribution pattern of LDs that were primarily located in the cytoplasm (Fig. 2C).

### *Nb* infection affected LD size and *plin1* expression

**Fig 3:**
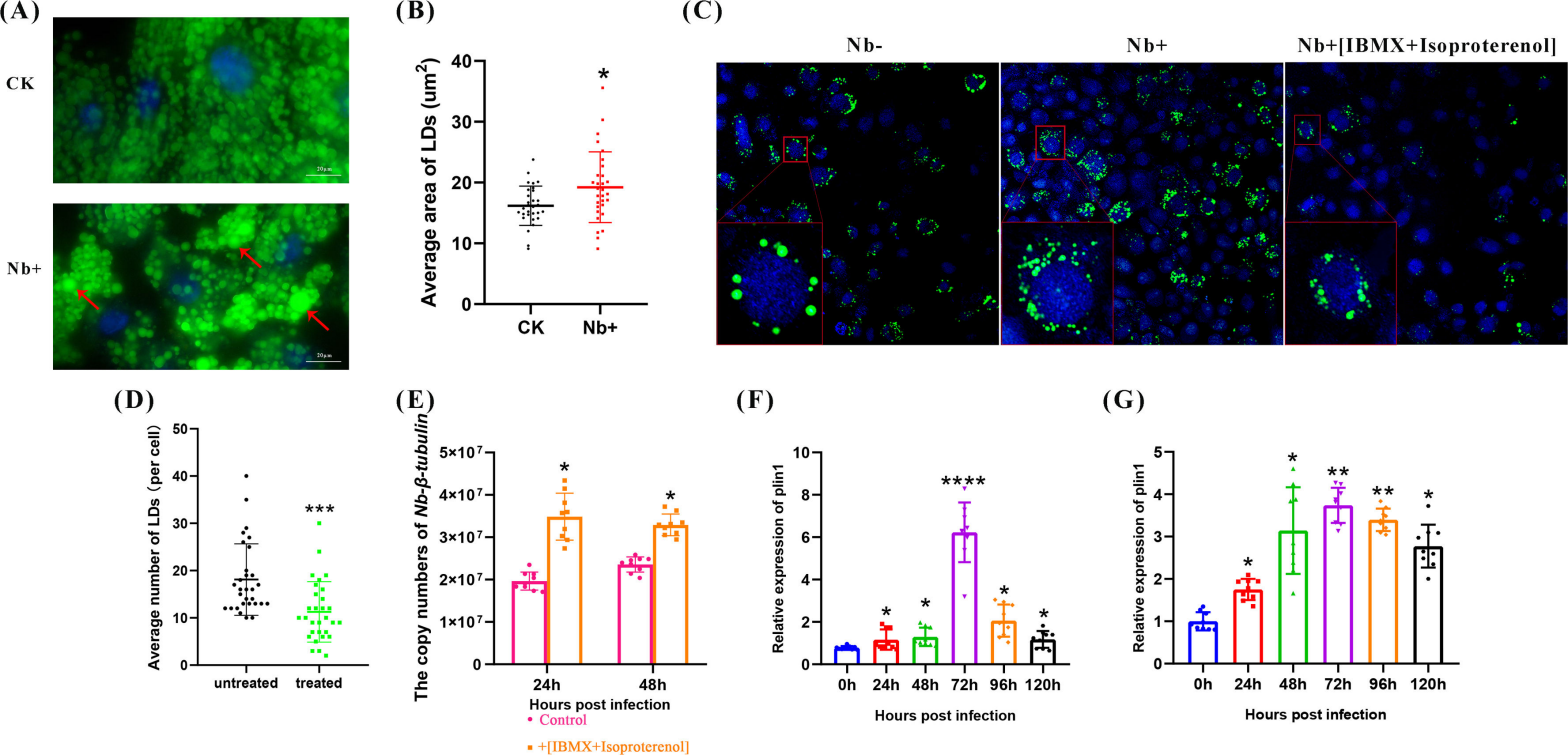
Effects of *Nb* infection on lipid droplet size and *plin1* expression. (**A**) Fat body of uninfected and infected groups; CK and Nb +were collected at 72 h p.i. Samples were stained with BODIPY 493/503 (green) and DAPI (blue) for LD visualization; scale bar = 20 µm. (**B**) Statistics of LD size in the fat body from CK and Nb +silkworms. (**C**) IBMX and isoproterenol inhibit LD synthesis. BmN cells were stained with BODIPY 493/503 (green) and DAPI (blue) for LD visualization; scale bar = 20 µm. (**D**) Average number of LDs per cell in the IBMX- and isoproterenol-untreated and -treated cells. (**E**) Copy number of *Nb* after IBMX and isoproterenol treatment. (**F and G**) *plin1* expression in the fat body and midgut of *Nb-*infected silkworms. Error bars represent mean ± SD. All the experiments were repeated three times. One-way ANOVA or *t*-test, *<*I>P* < 0.05, **P < 0.01, and ****P < 0.0001. ns, not significant.

Various pathogens have been found to promote the formation of LDs (27–29). To investigate the changes in LDs after *Nb* infection, the fat bodies from *Nb-*infected and -uninfected larvae were collected and stained to observe the morphology of the LDs. The results showed that LDs in the *Nb-*infected group were larger than those in the control group, suggesting that *Nb* infection may induce LD accumulation in silkworms (Fig. 3A through D). Previous studies have shown that LDs are associated with infections by pathogens, including viruses, bacteria, fungi, and protists (30). To verify the effect of LDs on *Nb* reproduction, we initially confirmed that the formation of LDs could be significantly inhibited in BmN cells by IBMX and isoproterenol, which are inhibitors of LD formation (31). Also, the copy number of *Nb* was significantly higher in the inhibitor-treated groups at the early stage [24 hrs post-infection (h p.i.)] but was not significantly different at 48 h p.i. (Fig. 3E), suggesting that intracellular LDs may play a role in the early stages of *Nb* infection. To determine whether *plin1* is associated with *Nb* infection, the expression of *plin1* during *Nb* infection in the fat body and midgut was detected. The results showed that p*lin1* was slightly increased from 24 to 48 h p.i., massively increased 72 h p.i., and then decreased dramatically at 96 to 120 h p.i. in the fat body (Fig. 3F). In the midgut tissues, *plin1* expression was increased significantly from 24 to 72 h p.i. and then decreased slowly at 96 h p.i. (Fig. 3G). The difference between infected fat bodies and the midgut may indicate that plin1 in the midgut is more sensitive to Nb infection. These results suggest a correlation between *Nb* infection, LD accumulation, and *plin1* expression.

### *plin1* promotes LD accumulation and inhibits *Nb* proliferation

**Fig 4:**
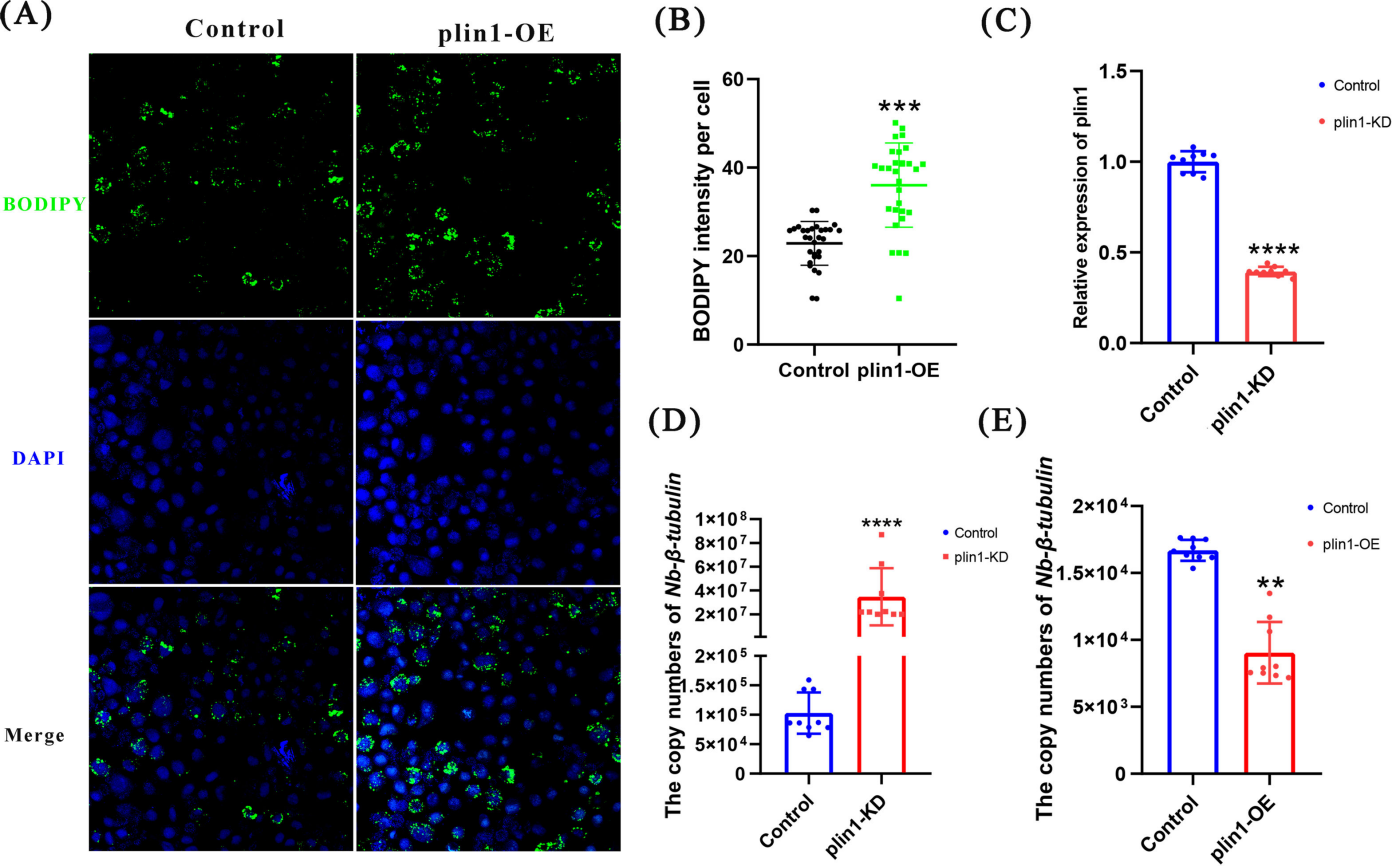
*plin1* affects the proliferation of *Nb*. (**A**) Changes in the number of lipid droplets (LDs) after overexpression of *plin1*. Samples were stained with BODIPY 493/503 (green) and DAPI (blue) for LD visualization; scale bar = 50 µm. (**B**) BODIPY intensity per cell. (**C**) *plin1* expression levels decreased significantly after RNAi interference. (**D**) Copy number of *Nb* after *plin1* knockdown. (**E**) *Nb* copy number after overexpression of *plin1*. All the experiments were repeated three times. Error bars represent mean ± SD. *t*-test, **<*I>P* < 0.01, ***P < 0.001, and ****P < 0.0001.

As LD surface proteins, PLINs maintain LD homeostasis. Studies have shown that PLIN1 can prevent lipases from approaching LDs, and when the energy is deficient, PLIN1 is phosphorylated to recruit lipase HSL, resulting in lipolysis (32). *Plin1* expression leads to LD clustering (33). To investigate the effects of *plin1* expression on the LDs in silkworm cells, we overexpressed *plin1*. A significant number of BODIPY-stained LDs were observed in *plin1*-overexpressing cells, indicating that *plin1* promotes lipid synthesis or inhibits LD degradation (Fig. 4A and B). To confirm the relationship between *plin1* and *Nb* proliferation, *plin1* in BmN cells was knocked down (KD) by siRNAs. The results showed that the siRNA effectively reduced the expression of *plin1* in the cells, and the copy number of *Nb* significantly increased after *plin1* KD, indicating that reduction of *plin1* favors proliferation of *Nb* (Fig. 4C and D). In contrast, *plin1* overexpression caused a decrease in *Nb* copy number (Fig. 4E). The results of the overexpression and KD experiments confirmed that *plin1* inhibits the proliferation of *Nb*. There are two perilipin genes in *Drosophila,* and PLIN1 plays a major role in the regulation of lipid metabolism, whereas PLIN2 plays a compensatory function when PLIN1 was missing (12). The NCBI database contains another gene known as *perilipin* of *B. mori* (*plin2*, accession number NM_001145332). We also investigated the effects of *plin2* overexpression and knock down on *Nb* replication. *plin2* had a modest effect on *Nb* proliferation (Fig. S2), suggesting that *plin1* may play a dominant role in anti-*Nb* activity, whereas *plin2* may act as a helper to *plin1*.

### *plin1* promotes JAK-STAT immune pathway

**Fig 5:**
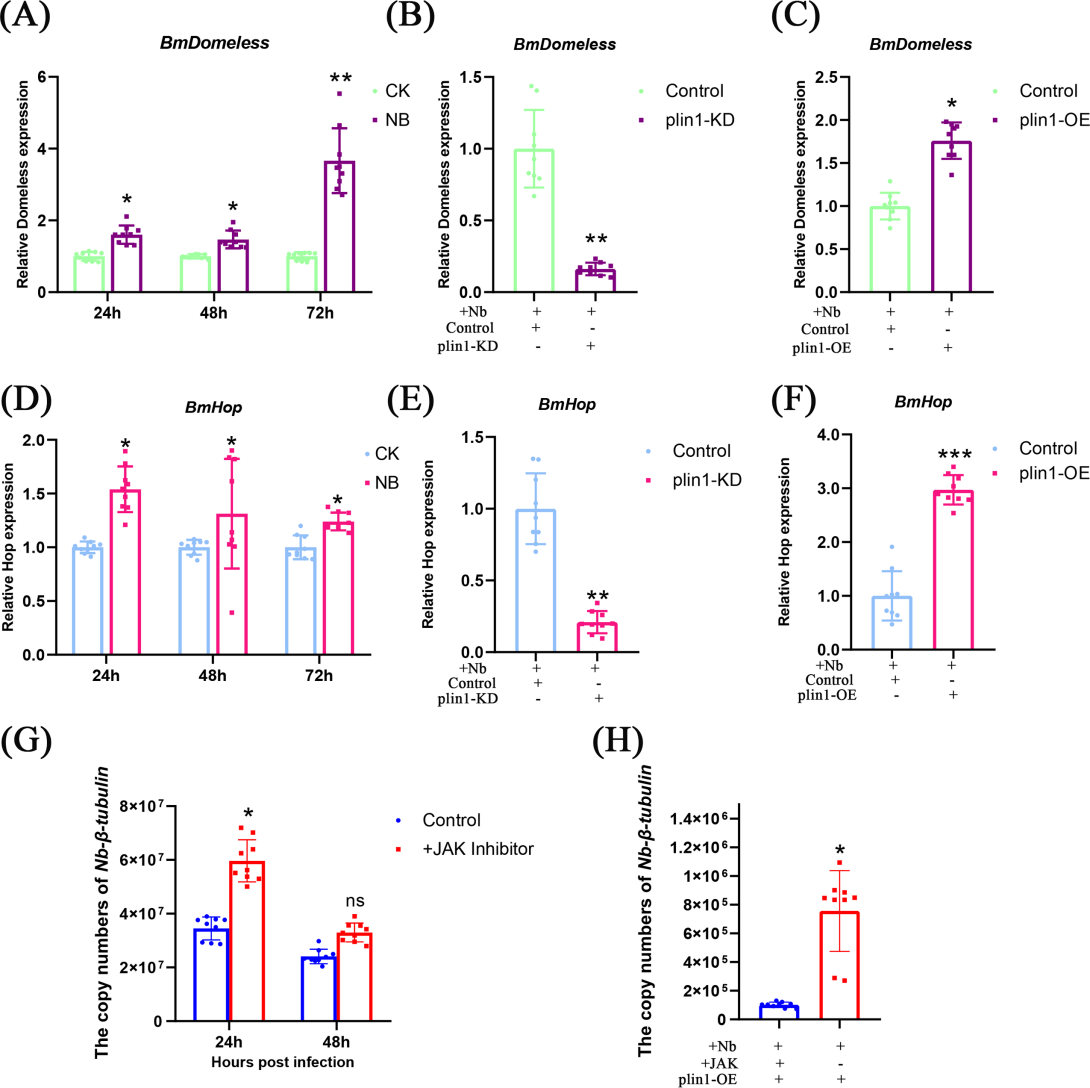
*plin1* enhances the anti-*Nb* effect of the JAK-STAT pathway by promoting *Domeless* and *Hop* expression. (**A, D**) Quantification of *Domeless* and *Hop* expression by RT-qPCR in BmN cells at 24, 48, and 72 hrs post-*Nb* infection. (**B, E**) Quantification of *Domeless* and *Hop* expression after knockdown of *plin1* in BmN cells. BmN cells transfected with dsRNA-eGFP were used as a negative control. (**C, F**) Quantification of *Domeless* and *Hop* expression after overexpression of plin1 in BmN cells. BmN cells transfected with the empty vector were used as a negative control. (**G**) Quantification of *Nb* copy number by RT-qPCR after treatment with the JAK inhibitor at 24 and 48 h p.i. in BmN cells. BmN cells treated with DMSO were used as the negative control. (**H**) Quantification of *Nb* copy number by RT-qPCR after treatment with the JAK inhibitor and overexpression of *plin1* in BmN cells. BmN cells treated with DMSO were used as the negative control. All the experiments were repeated three times. Error bars represent mean ± SD. One-way ANOVA or *t*-test, *<*I>P* < 0.05, **<*I>P* < 0.01, and ***<*I>P* < 0.001. ns, not significant.

Previous studies have shown that LDs are the primary organelles for lipid storage in eukaryotic cells and that they also possess antimicrobial properties (34). In *Drosophila*, *perilipin* expression is associated with bacterial-induced activation of the IMD pathway (35). Therefore, we hypothesized that *plin1* may inhibit *Nb* proliferation by affecting the immune pathway in silkworms. To investigate this, we examined the effects of *plin1* expression on key genes of the IMD, JAK-STAT, and TOLL pathways in silkworms. The results showed that among the 11 tested genes, only *Domeless* and *Hop* from the JAK-STAT pathway were significantly upregulated (Fig. S3 and S4). RT-qPCR analysis demonstrated a significant increase in the expression of *Domeless* and *Hop* following Nb infection (Fig. 5A and D). To verify the effect of *plin1* on *Domeless* and *Hop*, we examined their expression in *plin1*-overexpressing and KD cells. The results showed that the expression of *Domeless* and *Hop* decreased after *plin1* KD (Fig. 5B and E) and increased after its overexpression (Fig. 5C and F). To test whether *Domeless* or *Hop* has a direct effect on *Nb* proliferation, we also examined the influence of Nb copy number after *Domeless* or *Hop* RNAi. The result showed that the number of *Nb* copies increases significantly when *Domeless* or *Hop* was decreased by siRNAs (Fig. S6). Furthermore, we inhibited the JAK-STAT pathway with a JAK inhibitor. The results showed that blocking the JAK-STAT pathway led to an increase in *Nb* proliferation in both normal cells and *plin1*-OE cells (Fig. 5G and H), indicating that the JAK-STAT pathway is involved in the process of resistance against *Nb* proliferation in silkworms.

## DISCUSSION

LDs are dynamic and ubiquitous organelles found in all cells ranging from prokaryotes to eukaryotes. In addition to modulating lipid and energy homeostasis, LDs respond to ER stress, oxidative stress, protein maturation, and turnover (36). LD accumulation in the cytoplasm of distinct host cell types has been reported after infection with *Plasmodium berghei* (37, 38), *Trypanosoma cruzi* (39–41), *Toxoplasma gondii* (42–44), and *Leishmania major* (45, 46). For example, protozoan parasites (such as *Leishmania amazonensis* and *Plasmodium chabaudi*) are able to induce LD genesis in non-immune and immune cells that not only accumulate in the host cytoplasm but also relocate around and move into parasitophorous vacuoles (47, 48). Salmonellosis induces a time-dependent increase in LD formation in macrophages, and the inhibition of diacylglycerol O-acyltransferase 1 and cytosolic phospholipase A2 significantly reduces intracellular bacterial proliferation (49). Chronic hepatitis C virus (HCV) infection also triggers lipid accumulation in human liver-derived cells (50). In the present study, we found that *Nb* infection caused the accumulation of LDs in the fat body of silkworms, which is consistent with most pathogenic infections, indicating that the accumulated LDs are an important site of antagonism between the infectious agent and the host.

As LD surface proteins, PLINs regulate the formation and decomposition of LDs (51, 52). Perilipin is also closely associated with pathogenic infections. PLIN1 can respond to IMD activation and alter morphological changes in LDs to mitigate the immune response against bacterial infections in *Drosophila* (35). In mice infected with *Pseudomonas aeruginosa*, upregulation of PLIN2 leads to the accumulation of LDs in lung tissues, which ultimately triggers the release of COX-2-mediated anti-inflammatory cytokines to achieve immune effects (53). In hepatocytes, PLIN2 is necessary for the translocation of HCV nuclei and NS5A proteins to LDs and the formation of functional, low-density HCV viral particles (54). Perilipin KD in shrimp inhibited the proliferation of *Vibrio parahaemolyticus* by increasing the production of reactive oxygen species (55). *Mycobacterium leprae* infection induces perilipin expression to facilitate lipid accumulation within the phagosome and creates a suitable environment for intracellular survival within macrophages (56). In a parasitic infection model, increased levels of PLIN2 were observed in the liver of pregnant mice infected with *Plasmodium berghei* NK65 (57). Studies have suggested that PLINs interact directly with cytosolic bacteria. The *Dictyostelium* homolog of mammalian perilipin binds to the cytosolic bacterium *Mycobacterium marinum* (58). In the current study, we found that *plin1* inhibited the replication of *Nb*. All data confirmed a complex relationship between PLINs and infectious diseases.

Previous transcriptomic data have indicated that *Nb* infection activates immune pathways such as IMD, Toll, and JAK-STAT in silkworms (25). We found that *Domeless* and *HOP* from the JAK-STAT pathway were induced by *Nb* infection or *plin1* expression, suggesting that *plin1* may inhibit *Nb* replication by activating the JAK-STAT pathway. Blocking the JAK-STAT pathway with an inhibitor also confirmed that the JAK-STAT pathway is utilized by silkworms to inhibit *Nb* proliferation. Previous studies have shown that the JAK-STAT pathway is a functional approach to the mammalian interferon system (25, 59) and plays an important role in fat body function and the regulation of lipid and glucose metabolism (60). In insects, the JAK-STAT pathway promotes the production of other proteins, including stress-response proteins and cytokines in the fat body. Furthermore, JAK-STAT signaling stimulates the production of antimicrobial peptides to resist infection (61). *Domeless*, first identified in *Drosophila melanogaster*, is a unique receptor involved in the invertebrate JAK/STAT pathway (3, 62). *Domeless* KD significantly increases the replication of *Drosophila melanogaster* sigma virus (63). Tu *et al.* found that *Domeless* KD significantly increased the pathogenicity of *Beauveria bassiana* against the leaf beetle *Plagiodera versicolora* (64). In the shrimp *Marsupenaeus japonicus*, *Vibrio* infection induced the expression and secretion of peroxiredoxin 4, which could bind to *Domeless* to abrogate the activation of the JAK/STAT pathway (65). In silkworms, *Domeless* overexpression upregulates the expression of JAK/STAT pathway-related genes, promotes proliferation, and inhibits apoptosis (66). Hop is a tyrosine kinase in the JAK-STAT pathway, which has been found to autophosphorylate and phosphorylate *Domeless* in *Drosophil*a. A point mutation in *Drosophila* Hop causes constitutive activation of the JAK/STAT pathway (67) and mutant *Drosophila* for Hop ws more susceptible to viral infection compared to the wild-type (68). Dengue virus infection levels can increase in mosquitoes after Hop interference (69). In this study, we revealed that *Nb* infection induced massive expression of *plin1*, followed by activation of the JAK-STAT pathway by promoting the expression of *Domeless* and *Hop*. It is worth noting that *plin1* may not directly act as a transcription factor to promote the expression of *Domeless* and *Hop*, as its effect on these genes may be mediated by other proteins. As antimicrobial peptides responded to *Nb* infection and the JAK-STAT signaling pathway may regulate their expression, we investigated the effects of plin1 on silkworm antimicrobial peptides (70). The results showed the expression of gloverin 2–4 was significantly increased after *plin1* overexpression (Fig. S5), suggesting the ultimate effectors of *plin1* against *Nb* proliferation may be gloverins.

**Fig 6:**
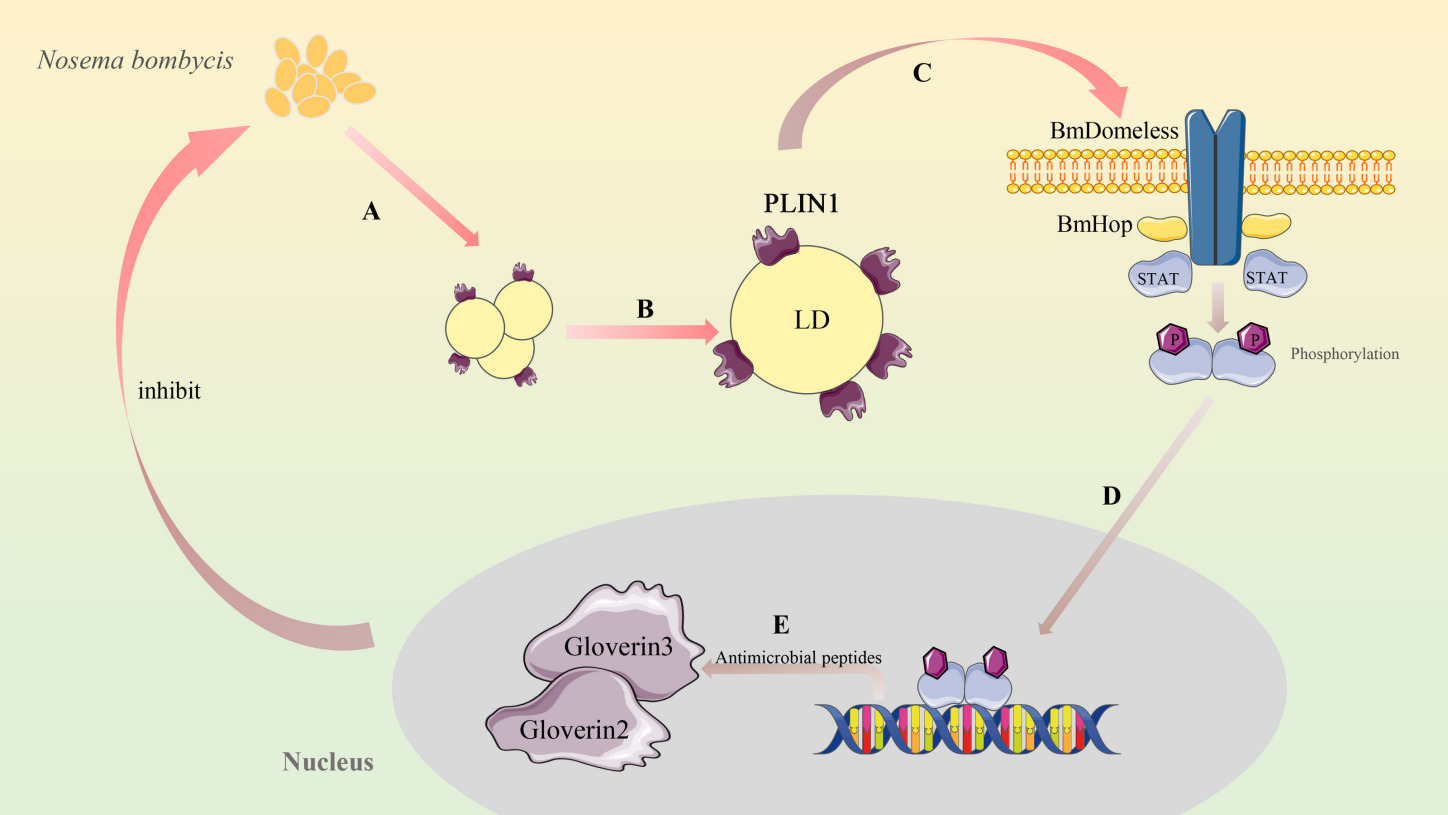
Overview of plin1 inhibiting *Nb* proliferation in silkworms. (**A-B**) Accumulation of lipid droplets and expression of *plin1* were induced by *Nb* infection. (**C**) Expression of *Domeless* and *Hop* is promoted by plin1, followed by activation of the JAK-STAT immune pathway. (**D-E**) plin1 promotes the expression of antimicrobial peptides gloverin 2 and gloverin 3, which inhibit the proliferation of *Nb*.

In conclusion, the present study showed that silkworm *plin1* inhibits *Nb* cell proliferation by promoting the JAK-STAT pathway through the increased expression of *Domeless* and *Hop*. The hypothetical anti-*Nb* pathway of *plin1* in silkworms can be summarized as follows: when the signal of *Nb* infection is transmitted to the nucleus, the transcription of *plin1* is activated, which not only promotes the aggregation of LDs but also enhances the expression of *Domeless* and *Hop*. The activation of JAK-STAT promotes the expression of antimicrobial peptides, such as gloverins, which inhibit *Nb* proliferation (Fig. 6). These results provide new insights into the complex relationship between microsporidian pathogens, LD surface proteins, and insect immunity.

## MATERIALS AND METHODS

### Silkworm culture and *Nb* challenge

Silkworm strain P50 was provided by the Sericultural Research Institute of the Chinese Academy of Agricultural Sciences, Zhengjiang. The larvae were fed with fresh mulberry leaves twice a day. The oral infection of *Nb* to silkworm was performed as follows: the first day fifth instar larvae were divided into two groups. For the *Nb* treatment group, fresh mulberry was immersed in 1 × 10^7^ spores per mL of *Nb* solution, air-dried at room temperature, and then fed to the silkworms for 4 hrs. Control larvae were fed dried mulberry immersed in distilled water. Tissues from the midgut, testis, fat body, and so on were collected from the third day fifth instar larva of *B. mori* P50.

### Total RNA extraction and genomic DNA extraction

The RNA isolation total RNA extraction reagent (R401-01, Vazyme, Nanjing, China) was used for total RNA isolation from silkworm tissues or BmN cells. mRNA was reverse-transcribed into cDNA with HiScript IIQ RT SuperMix for qPCR (R222-01, Vazyme, Nanjing, China). For the copy number of *Nb* detections, genomic DNA from infected silkworms or BmN cells was extracted using the DNA Kit (Code No.9765, TaKaRa MiniBEST Universal Genomic DNA Extraction Kit Ver.5.0, Japan) according to the instruction manual.

### Sequence and phylogenetic analysis

The characteristic domains and sequence of *plin1* were obtained from the National Center for Biotechnology Information (NCBI) databases. MEGA software (71) (version 11.0.10) was used to construct the multiple sequence alignment and phylogenetic tree using the maximum likelihood (ML) method and genes from different species. The multiple sequence alignment was constructed based on the amino acid sequence of perilipin proteins. The phylogenetic tree was constructed based on the gene sequence of perilipin (the species accession numbers for comparison are shown in Table S2).

### RT-qPCR

The samples from silkworm and BmN cell were collected at different times of injection with *Nb* or ddH_2_O (control group). After RNA extraction and cDNA synthesis, RT-qPCR was performed using ChamQ SYBR qPCR Master Mix (Q341-02, High ROX Premixed, Vazyme, Nanjing, China). The RT-PCR program was as follows: one cycle of pre-denaturation for 30 s at 95°C, 40 cycles of 95°C for 10 s, and 60°C for 30 s. The relative mRNA level of genes was determined by the 2**^-ΔΔCt^** method, using *BmGADPH* as an internal reference. All the primers are listed in Table S1. *Nb-tubulin* gene-specific primers were used to amplify the *tubulin* gene of *Nb*, as indicator of the genome copy number (26). A standard curve described by y = −2.3327 x +38.202 (R^2^ = 0.9557) was used to evaluate the copy number of *Nb*. Genomic DNA from infected silkworms or BmN cells (100 ng of each DNA sample) was detected with the following program: one cycle of pre-denaturation for 30 s at 95°C, 40 cycles of 95°C for 5 s, and 60°C for 30 s.

### Gene cloning and vector construction

According to the nucleotide sequence of BGIBMGA013593-PA in SilkDB 3.0, primers (Table S1) were designed to amplify *plin1* in silkworm cDNA by PCR. The PCR program was 95°C for 3 min, 35 cycles of 95°C for 15 s, 50°C for 15 s, and 72°C for 1 min, with a final stage of 72°C for 5 min. The *plin1* gene was cloned into the plasmid pIZ-mCherry to obtain pIZ-mCherry-plin1 (72). The *plin1* gene was cloned into the plasmid vBac-eGFP to obtain vBac-eGFP-plin1. *EcoRI* and *XhoI* restriction sites were used to clone into both of the abovementioned vectors. All the plasmid constructs were verified by sequencing.

### RNAi and cell transfection

RNA interference (RNAi) was used to knockdown the expression of *plin1*. The siRNAs are listed in Table S1. BmN cells were seeded into a 12-well culture plate and were maintained in TC 100-insect medium (A2017,9010, RanReac AppliChem, Germany) with 10% DMEM (10099–141C, Gibco, Shanghai, China) and 1% penicillin–streptomycin solution (E607011, 100X, BBI Life Sciences, Sangon Biotech, shanghai, China) overnight. One microgram pIZ-mCherry-plin1 or 40 pmol siRNA-plin1 was transfected into cells using the Lipo 8000 transfection reagent (C0533, Beyotime, Shanghai, China) according to the instructions. Forty-eight hours after transfection, the RNAi efficiency was detected with RT-qPCR.

### Localization and Western blotting analysis

After the vBac-eGFP-plin1 plasmids were transfected into BmN cells for 80% fluorescence efficiency, the cells are collected, the culture medium is removed by centrifugation, and then lysed with RIPA (P0013B, Beyotime Biotechnology, Shanghai, China) at 4°C for 30 min. Then, we added 2ⅹ protein loading buffer at 100°C for 10 min (C508319, BBI, Sangon Biotech, Shanghai, China). Protein samples were separated by 12% sodium dodecyl sulfate-polyacrylamide gel electrophoresis (SDS-PAGE) and were transferred onto a PVDF transfer membrane (Immobilon-P^SQ^ Transfer Membrane, Merck Millipore, Ireland). After blocking the membrane with 5% milk (A600669, NON-Fat Powdered Milk, Sangon Biotech, Shanghai, China) dissolved in a PBST buffer (G101-01, Vazyme, Nanjing, China) containing 1‰ Tween-20 (A600560, Diamond, Sangon Biotech, Shanghai, China) at room temperature for 1 h, the anti-eGFP mouse monoclonal antibody 1:2000 (D199989, BBI, Sangon Biotech, Shanghai, China) was used to incubate the membrane for 2 hours at room temperature. Then, the membranes were incubated with goat anti-mouse IgG (H + L) HRP-conjugated secondary antibody 1:5000 (A30333, MULTI SCIENCES, China) for 1 h, after which Tanon High-sign ECL Western Blotting Substrate was used for detection with the Tanon machine (Tanon, Shanghai, China).

### LD staining and microscopy

The fat body or BmN cells with pIZT-mCherry-plin1 was collected and fixed in 4% paraformaldehyde fix solution (E672002, BBI, Sangon Biotech, Shanghai, China) for 10 min on ice. Fat bodies were then rinsed twice with PBS and incubated in PBS containing 1 µg/mL of BODIPY 493/503 (ajci70160, Amgicam, Shanghai, China) for 30 min, 1 µL/mL DAPI (D-9564, SIGMA) was added to stain nuclei for 5 min. After staining, fat bodies were rinsed three times with PBS. Stained samples were mounted in 80% glycerol (A100854, Diamond, Sangon Biotech, Shanghai, China) for microscopy analysis (73). All images were taken using an inverted fluorescence microscope and cellSens Standard software (OLYMPUS, BX53M, Japan). To quantify LD size and number in each cell form 30 fat body cells, the ImageJ analysis software was used to measure the size and number (74).

### Treatment of cells with the LD inhibitor and JAK inhibitor

BmN cells were incubated in a medium containing 0.5–1mM IBMX (HY-12318, MCE, Nanjing, China) and 1 mM isoproterenol (HY-B0468, MCE, Nanjing, China) for 24 h and 48 h, respectively. When these two chemicals were used in combination, LDs were found to disperse in cells (31). BmN cells were treated with 0.5–1 mM JAK inhibitor (HY-18300, Filgotinib, MCE, Nanjing, China) to inhibit JAK synthesis (75). The copy number of *Nb* was detected after using the LD inhibitor and JAK inhibitor with RT-qPCR.

### Statistical analysis and reproducibility

Results are expressed as the mean ± standard deviation (SD). All statistical analyses were performed using the GraphPad Prism 8.0 software. The statistical significance was represented by *P* values of > 0.05, <0.05, <0.01, <0.001, or <0.0001 based on the *t*-test.

## SUPPLEMENTAL MATERIAL

Supplemental figures spectrum.03671-23-s0001.pdf Fig. S1-S6.

Table S1 spectrum.03671-23-s0002.docx The names and sequences of the oligoes used in the manuscript.

Table S2 spectrum.03671-23-s0003.docx The accession numbers of perilipins from different species.

ASM does not own the copyrights to Supplemental Material that may be linked to, or accessed through, an article. The authors have granted ASM a non-exclusive, world-wide license to publish the Supplemental Material files. Please contact the corresponding author directly for reuse.
