# Supplementary material for: Perilipin1 inhibits Nosema bombycis proliferation by promoting Domeless- and Hop-mediated JAK-STAT pathway activation in Bombyx mori
Source: Microbiol Spectr. 2024 May 1;12(6):e03671-23. doi: 10.1128/spectrum.03671-23 (PMC11237581; doi:10.1128/spectrum.03671-23)
Supplement: Supplemental figures — Fig. S1-S6. [file spectrum.03671-23-s0001.pdf]

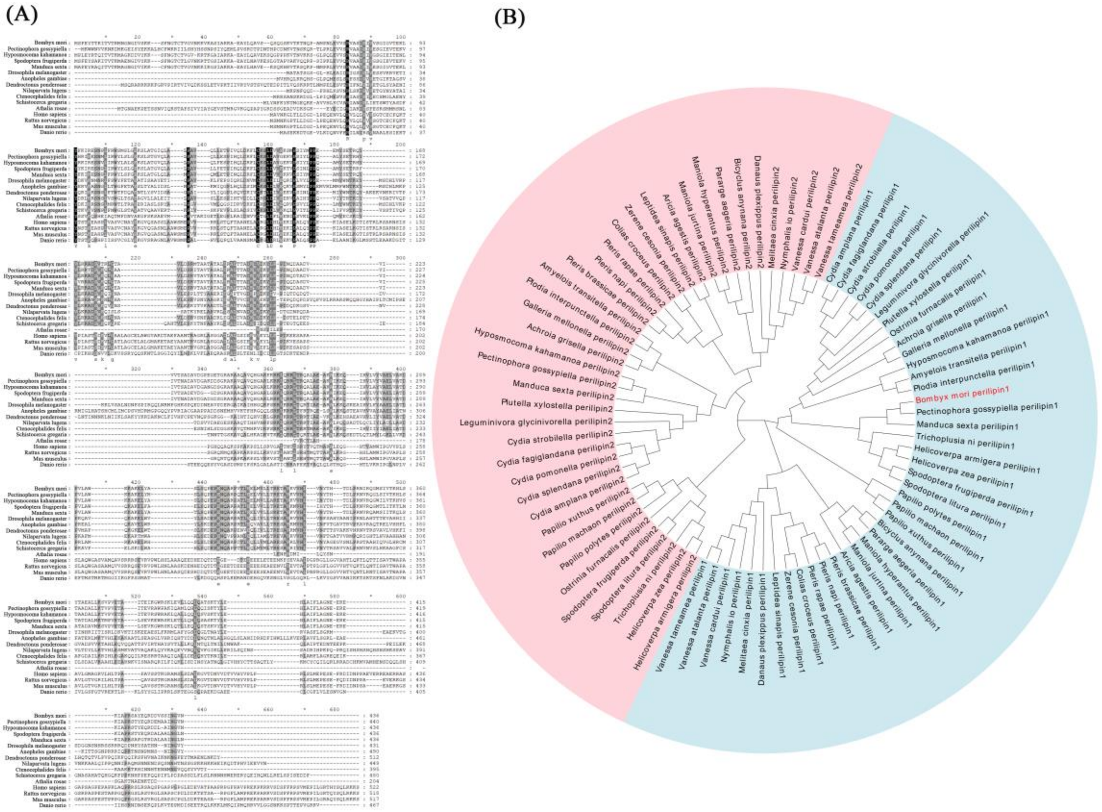

FIGURE S1. Sequence alignment, and evolutionary tree construction of plins. (A) The alignment of plin1 from the silkworm and other insect, including invertebrate and vertebrate species: *Pectinophora gossypiella*, *Hyposmocoma kahamanoa*, *Spodoptera frugiperda*, *Manduca sexta*, *Drosophila melanogaster*, *Anopheles gambiae*, *Dendroctonus ponderosae*, *Nilaparvata lugens*, *Ctenocephalides felis*, *Schistocerca gregaria*, *Athalia rosae*, *Homo sapiens*, *Rattus norvegicus*, *Mus musculus*, *Danio rerio* (The Protein ID of each sequence is shown in Table S2). (B) Phylogenetic analysis of plins from the silkworm and other Lepidoptera insect. The phylogenetic tree was generated based on the plins nucleotide sequences (The Gene ID of each sequence is shown in Table S2). The tree was color-coded with blue (perilipin1) and pink (perilipin2).

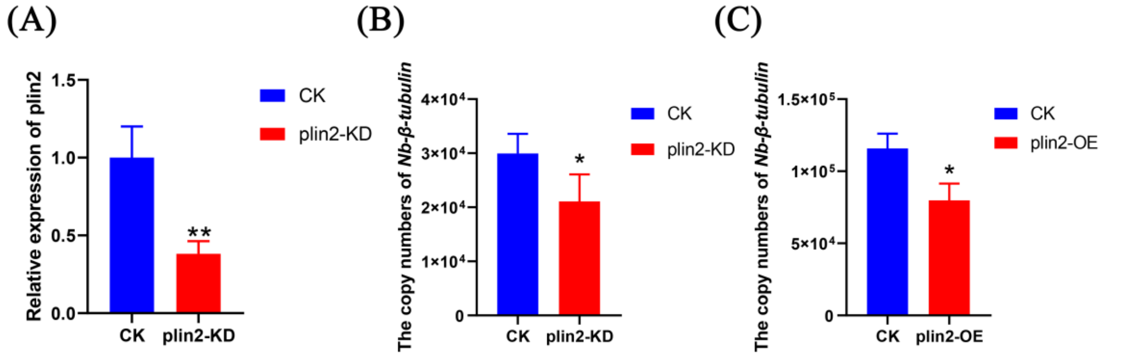

FIGURE S2. the effect of *plin2* on the proliferation of *Nb*. (A) *plin2* expression levels decreased significantly after RNAi interference. (B) Copy number of *Nb* after *plin2* knockdown. (C) *Nb* copy number after overexpression of *plin2*. Error bars represent mean ± SD. * $p < 0.05$, ** $p < 0.01$.

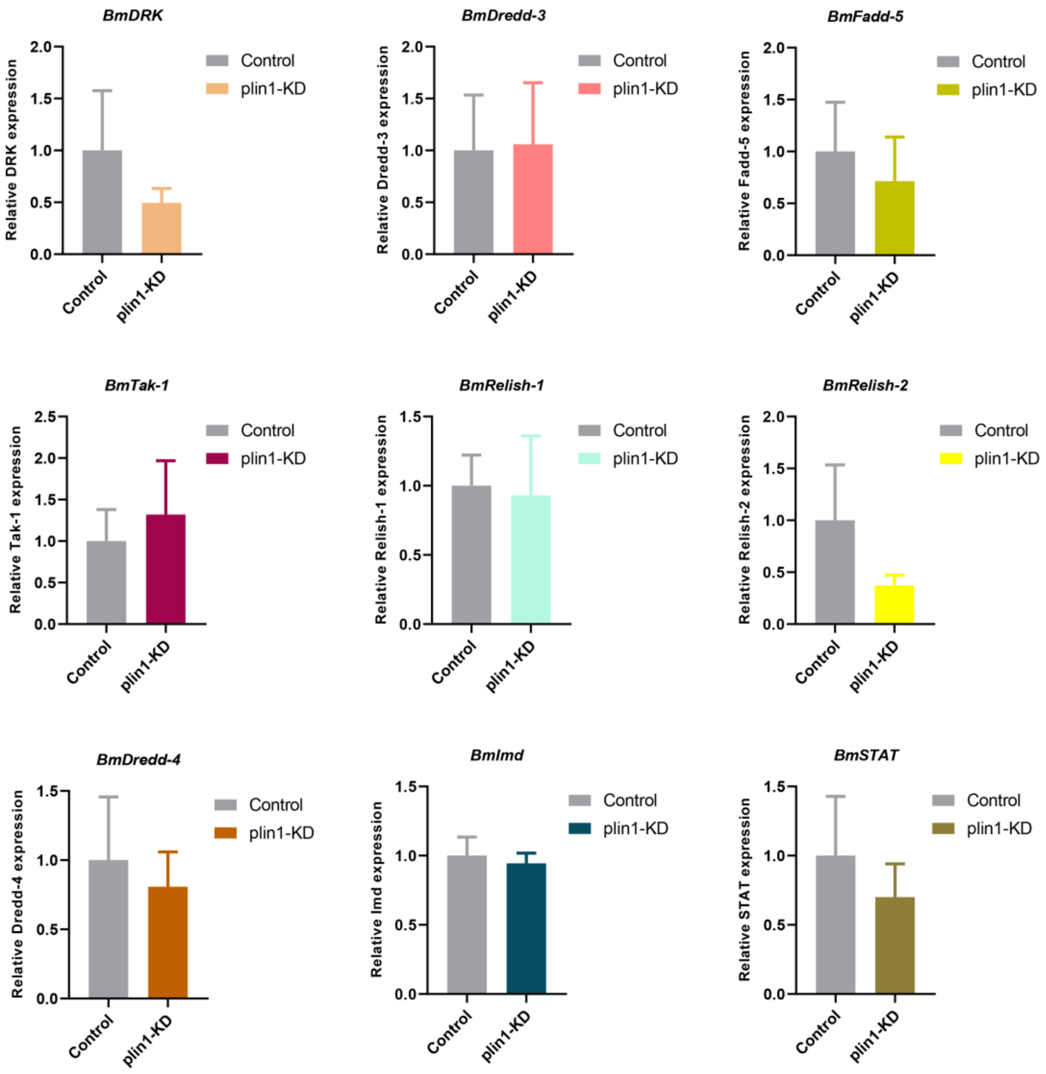

FIGURE S3. The expression of immune pathway related genes which have no significant changes after *plin1* knock down including *BmDRK*, *BmDredd*, *BmFadd*, *BmTak*, *BmRelish*, *BmImd*, *BmSTAT*.

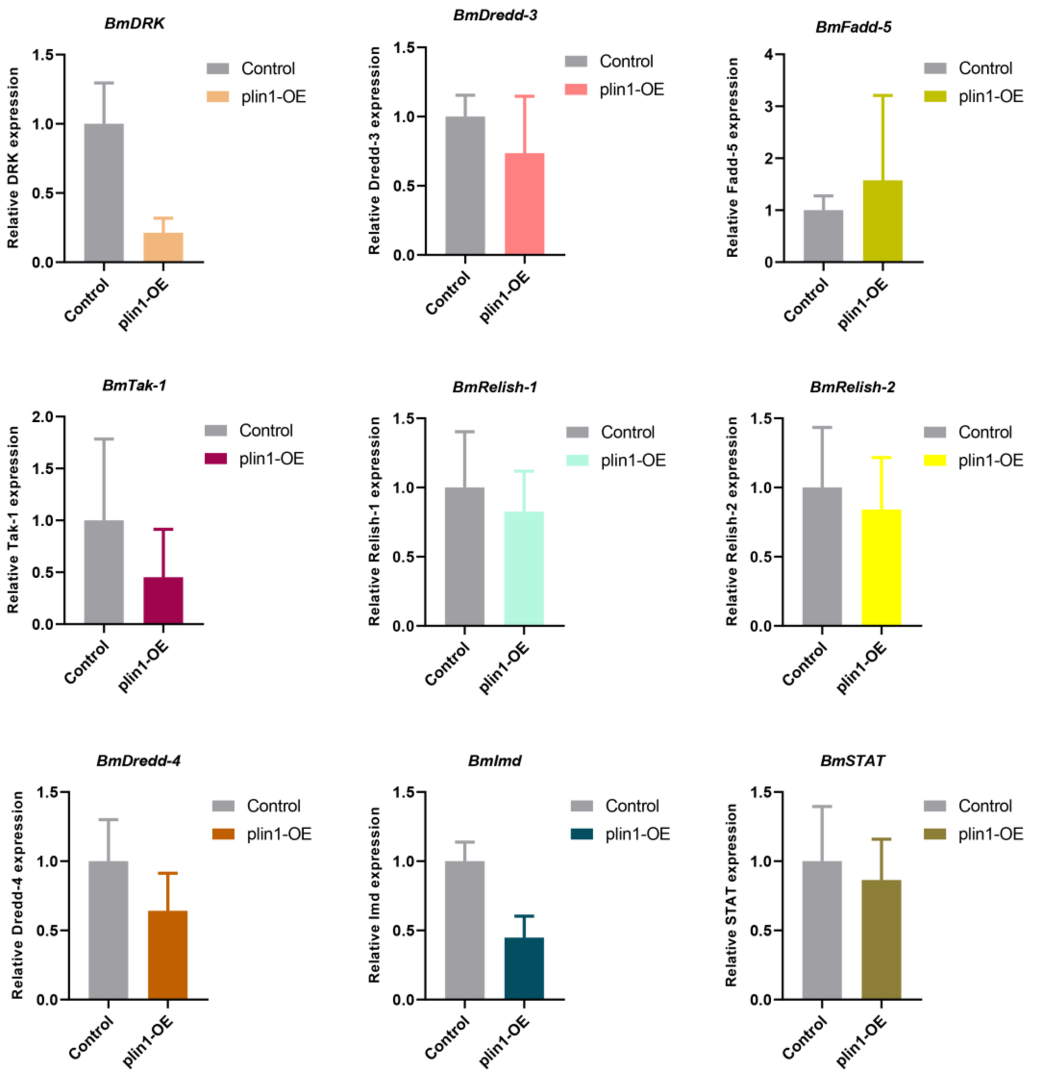

FIGURE S4. The expression of immune pathway related genes which have no significant changes after *plin1* overexpression including *BmDRK*, *BmDredd*, *BmFadd*, *BmTak*, *BmRelish*, *BmImd*, *BmSTAT*.

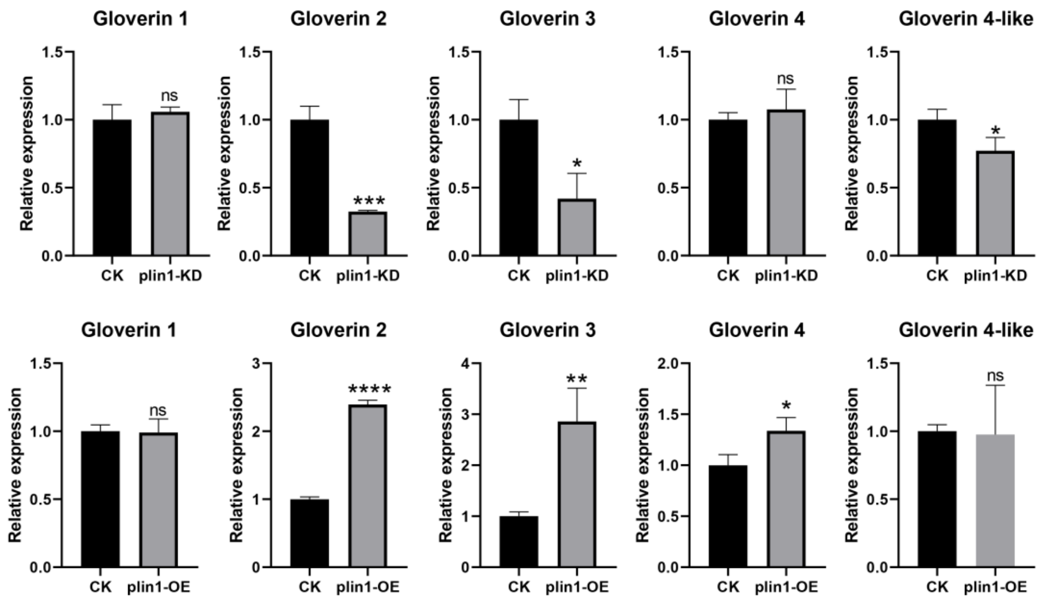

FIGURE S5. The effects of overexpression and knock down of *plin1* on the expression of antimicrobial peptide gloverins in BmN cells. The expression of gloverin2, 3 and 4-like was decreased after *plin1* knock down, no significant in gloverin1 and 4. Meanwhile, the expression of gloverin2, 3 and 4 was increased under *plin1* overexpression, no significant in gloverin1 and 4-like. Error bars represent mean ± SD. * $p < 0.05$, ** $p < 0.01$, *** $p < 0.001$, **** $p < 0.0001$, ns, no significant.

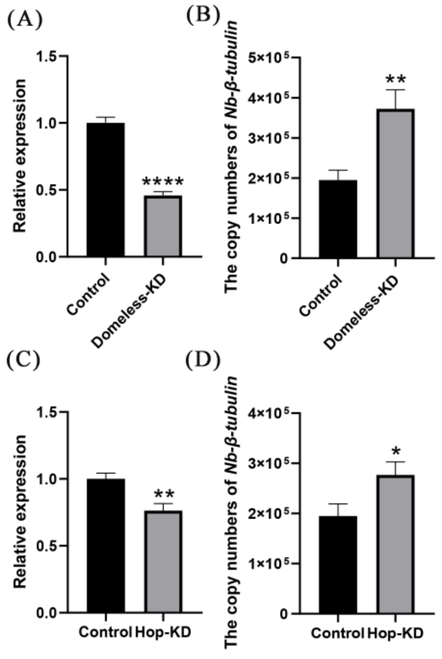

FIGURE S6. Effect of *Domeless* and *Hop* on Nb copy number after RNAi. (A, C) *Domeless* and *Hop* expression levels decreased significantly after siRNA transfected in BmN cells. (B, D) Copy number of Nb after *Domeless* and *Hop* knockdown. Error bars represent mean ± SD. t-test, * $p < 0.05$, ** p < 0.01, **** p < 0.0001. Error bars represent mean ± SD. * $p < 0.05$, ** $p < 0.01$, **** $p < 0.0001$, ns, no significant.
