# Supplementary material for: Perilipin1 inhibits Nosema bombycis proliferation by promoting Domeless- and Hop-mediated JAK-STAT pathway activation in Bombyx mori
Source: Microbiol Spectr. 2024 May 1;12(6):e03671-23. doi: 10.1128/spectrum.03671-23 (PMC11237581; doi:10.1128/spectrum.03671-23)
Supplement: Table S1 — The names and sequences of the oligoes used in the manuscript. [file spectrum.03671-23-s0002.docx]

Table S1. Oligos used

| Name | Sequence |
| --- | --- |
| plin1-F | CGGGATCCATGTCGTTCGAATACACC |
| plin1-R  plin2-F  plin2-R | CCCTCGAGATTGACACCGTTAATGG CGGAATTCATGGCGACAGAAGTAAGC  CCCTCGAGATTAGAAGGATCCACGGA |
| QRT- plin1-F | CGATCGTCGTGTCCGGTATT |
| QRT- plin1-R  QRT- plin2-F  QRT- plin2-R | CCGGGAGTGCTAGCTGTATG  CCCTCACGTGAACAAGGTGT  CAACCGGCGGTAGATAGTGG |
| QRT-BmGADPH-F | TTCATGCCACAACTGCTACA |
| QRT-BmGADPH-R | AGTCAGCTTGCCATTAAGAG |
| QRT-Nbβ-tubulin-F | TTCCCTTCCCTAGACTTCACTTC |
| QRT-Nbβ-tubulin-R | CAGCAGCCACAGTCAAATACC |
| Negative CK Sense | UUCUCCGAACGUGUCACGUTT |
| Negative CK Antisense | ACGUGACACGUUCGGAGAATT |
| plin1-siRNA Sense | GCGGUUCAACUCCUGGAAATT |
| plin1-siRNA-Antisense  plin2-siRNA Sense  plin2-siRNA Antisense  QRT-BmHOP-F  QRT-BmHOP-R  QRT-BmDomeless-F  QRT-BmDomeless-R  QRT-BmImd-F  QRT-BmImd-R  QRT-BmFadd5-F  QRT-BmFadd5-R  QRT-BmDredd3-F  QRT-BmDredd3-R  QRT-BmDredd4-F  QRT-BmDredd4-R  QRT-BmRelish1-F  QRT-BmRelish1-R  QRT-BmTak1-F  QRT-BmTak1-R  QRT-BmDRK-F  QRT-BmDRK-R  QRT-BmSTAT-F  QRT-BmSTAT-R  QRT-BmRelish2-F  QRT-BmRelish2-R | UUUCCAGGAGUUGAACCGCTT  GGAGUCGAAAUCCGUGGAUTT  AUCCACGGAUUUCGACUCCTT  GTCGGATACTTTGCCTTACC  ATTTTACTGCAACCGCAGAT  TTACACTGGCATTGAACACC  TGAATCACTAAGCACATCGG  AGCCGGAAGATGAGCATTTA  GTATCAATTCTGCCTTCGGTGA  GACACGGTGAGATTTTGAGG  TATCTTGTAGTCTTCGTGCG  TAGTATACTGGGCAACAGCA  AAATGCGGACCTGAGGATGA  AAGGTCGATTCACAACACAG  GTTCCATTTTCAGTTCGGGA  AATGCCGAAAACTGAGCTGC  GACCGAGTGTAGGTCCACG  TAGTCAAGGGCCTGTACAAA  GAAATCTACAGCTGTCGTCC  ATTTTACTGCAACCGCAGAT  TTCCCGTCTAATTCAGCTCT  TCGAGAGCAGAATATGGACC  GTACTGCATGTGGAAGTTCT  CGTCTGGGTCGTTGAAGAGT  AGTCAATGGATCAAGTACCTCAG |
