# Supplementary material for: Perilipin1 inhibits Nosema bombycis proliferation by promoting Domeless- and Hop-mediated JAK-STAT pathway activation in Bombyx mori
Source: Microbiol Spectr. 2024 May 1;12(6):e03671-23. doi: 10.1128/spectrum.03671-23 (PMC11237581; doi:10.1128/spectrum.03671-23)
Supplement: Table S2 — The accession numbers of perilipins from different species. [file spectrum.03671-23-s0003.docx]

Table S2. The accession numbers of perilipins from different species

| **Species Name** | **GenBank accession number** |
| --- | --- |
| Perilipin1 | mRNA |
| *Helicoverpa armigera* | XM_049836972.2 |
| *Danaus plexippus* | XM_062529373.2 |
| *Colias croceus* | XM_045640567.2 |
| *Pectinophora gossypiella* | XM_050024788.2 |
| *Manduca sexta* | XM_030273949.2 |
| *Maniola jurtina* | XM_045922358.2 |
| *Maniola hyperantus* | XM_034972524.2 |
| *Pararge aegeria* | XM_039894750.2 |
| *Bicyclus anynana* | XM_024092527.2 |
| *Nymphalis io* | XM_050493085.2 |
| *Vanessa atalanta* | XM_047675589.2 |
| *Vanessa cardui* | XM_047222446.2 |
| *Vanessa tameamea* | XM_026642724.2 |
| *Melitaea cinxia* | XM_045593468.2 |
| *Leptidea sinapis* | XM_050822866.2 |
| *Pieris brassicae* | XM_045658962.2 |
| *Pieris napi* | XM_047648732.2 |
| *Pieris rapae* | XM_045629206.2 |
| *Zerene cesonia* | XM_038363048.2 |
| *Aricia agestis* | XM_042232964.2 |
| *Papilio polytes* | XM_023282352.2 |
| *Cydia amplana* | XM_063528263.2 |
| *Cydia splendana* | XM_063779429.2 |
| *Cydia pomonella* | XM_062848242.2 |
| *Cydia strobilella* | XM_063684790.2 |
| *Cydia fagiglandana* | XM_063532228.2 |
| *Leguminivora glycinivorella* | XM_048243525.2 |
| *Achroia grisella* | XM_059298658.2 |
| *Galleria mellonella* | XM_052902207.2 |
| *Plodia interpunctella* | XM_053749532.2 |
| *Amyelois transitella* | XM_060946288.2 |
| *Ostrinia furnacalis* | XM_028305373.2 |
| *Helicoverpa zea* | XM_047269722.2 |
| *Spodoptera litura* | XM_022970524.2 |
| *Spodoptera frugiperda* | XM_035585222.2 |
| *Trichoplusia ni* | XM_026872394.2 |
| *Papilio machaon* | XM_045679280.2 |
| *Papilio xuthus* | XM_023322930.2 |
| *Hyposmocoma kahamanoa* | XM_026474384.2 |
| *Plutella xylostella* | XM_048633590.2 |
| Perilipin2 | mRNA |
| *Helicoverpa armigera* | XM_021339901.2 |
| *Danaus plexippus* | XM_032660066.2 |
| *Colias croceus* | XM_045640573.1 |
| *Pectinophora gossypiella* | XM_050014845.1 |
| *Manduca sexta* | XM_030174679.2 |
| *Maniola jurtina* | XM_045911367.1 |
| *Maniola hyperantus* | XM_034972829.1 |
| *Pararge aegeria* | XM_039894392.1 |
| *Bicyclus anynana* | XM_024092532.2 |
| *Nymphalis io* | XM_050493236.1 |
| *Vanessa atalanta* | XM_047675520.1 |
| *Vanessa cardui* | XM_047111538.1 |
| *Vanessa tameamea* | XM_026642695.1 |
| *Melitaea cinxia* | XM_045593086.1 |
| *Leptidea sinapis* | XM_050821813.1 |
| *Pieris brassicae* | XM_045660286.1 |
| *Pieris napi* | XM_047648496.1 |
| *Pieris rapae* | XM_022268579.2 |
| *Zerene cesonia* | XM_038364453.1 |
| *Aricia agestis* | XM_042112871.1 |
| *Papilio polytes* | XM_013282279.1 |
| *Cydia amplana* | XM_063518296.1 |
| *Cydia splendana* | XM_063779380.1 |
| *Cydia pomonella* | XM_061848397.1 |
| *Cydia strobilella* | XM_063691913.1 |
| *Cydia fagiglandana* | XM_063532125.1 |
| *Leguminivora glycinivorella* | XM_048140850.1 |
| *Achroia grisella* | XM_059198538.1 |
| *Galleria mellonella* | XM_026903899.3 |
| *Plodia interpunctella* | XM_053749996.1 |
| *Amyelois transitella* | XM_013337456.2 |
| *Ostrinia furnacalis* | XM_028317421.1 |
| *Helicoverpa zea* | XM_047170016.1 |
| *Spodoptera litura* | XM_022970375.1 |
| *Spodoptera frugiperda* | XM_035584749.2 |
| *Trichoplusia ni* | XM_026871407.1 |
| *Papilio machaon* | XM_045679188.1 |
| *Papilio xuthus* | XM_013312080.1 |
| *Hyposmocoma kahamanoa* | XM_026474420.1 |
| *Plutella xylostella* | XM_011554271.3 |
| Perilipin1 | Protein |
| *Pectinophora gossypiella*  *Hyposmocoma kahamanoa*  *Spodoptera frugiperda*  *Manduca sexta*  *Drosophila melanogaster*  *Anopheles gambiae*  *Dendroctonus ponderosae*  *Nilaparvata lugens*  *Ctenocephalides felis*  *Schistocerca gregaria*  *Athalia rosae*  *Homo sapiens*  *Rattus norvegicus*  *Mus musculus*  *Danio rerio* | XP 049870745.1  XP 026330169.1  XP 035441015.1  XP 030029809.1  NP 732904.2  XP 061498077.1  XP 048517264.1  XP 039278297.1  XP 026475145.1  XP 049832935.1  XP 012265027.2  NP 001138783.1  NP 001295074.1  NP 001106942.1  XP 021323484.1 |
